# Machine learning models for classification and identification of significant attributes to detect type 2 diabetes

**DOI:** 10.1007/s13755-021-00168-2

**Published:** 2022-02-09

**Authors:** Koushik Chandra Howlader, Md. Shahriare Satu, Md. Abdul Awal, Md. Rabiul Islam, Sheikh Mohammed Shariful Islam, Julian M. W. Quinn, Mohammad Ali Moni

**Affiliations:** 1 Department of CSTE, Noakhali Science and Technology University, Noakhali, Bangladesh; 2 Department of MIS, Noakhali Science and Techology University, Noakhali, Bangladesh; 3 Electronics and Communication Engineering Discipline, Khulna University, Khulna, Bangladesh; 4 School of Electrical, Computer and Telecommunications Engineering, Faculty of Engineering and Information Sciences, University of Wollongong, Wollongong, Australia; 5 Institute for Physical Activity and Nutrition, Deakin University, Geelong, Australia; 6 Bone Biology Division, Garvan Institute of Medical Research, Darlinghurst, NSW Australia; 7 School of Health and Rehabilitation Sciences, Faculty of Health and Behavioural Sciences, The University of Queensland, St Lucia, QLD 4072 Australia

**Keywords:** Diabetes, Classifiers, Feature selection sets, Prediction model, Machine learning models

## Abstract

Type 2 Diabetes (T2D) is a chronic disease characterized by abnormally high blood glucose levels due to insulin resistance and reduced pancreatic insulin production. The challenge of this work is to identify T2D-associated features that can distinguish T2D sub-types for prognosis and treatment purposes. We thus employed machine learning (ML) techniques to categorize T2D patients using data from the Pima Indian Diabetes Dataset from the Kaggle ML repository. After data preprocessing, several feature selection techniques were used to extract feature subsets, and a range of classification techniques were used to analyze these. We then compared the derived classification results to identify the best classifiers by considering accuracy, kappa statistics, area under the receiver operating characteristic (AUROC), sensitivity, specificity, and logarithmic loss (logloss). To evaluate the performance of different classifiers, we investigated their outcomes using the summary statistics with a resampling distribution. Therefore, Generalized Boosted Regression modeling showed the highest accuracy (90.91%), followed by kappa statistics (78.77%) and specificity (85.19%). In addition, Sparse Distance Weighted Discrimination, Generalized Additive Model using LOESS and Boosted Generalized Additive Models also gave the maximum sensitivity (100%), highest AUROC (95.26%) and lowest logarithmic loss (30.98%) respectively. Notably, the Generalized Additive Model using LOESS was the top-ranked algorithm according to non-parametric Friedman testing. Of the features identified by these machine learning models, glucose levels, body mass index, diabetes pedigree function, and age were consistently identified as the best and most frequently accurate outcome predictors. These results indicate the utility of ML methods in constructing improved prediction models for T2D and successfully identified outcome predictors for this Pima Indian population.

**Supplementary Information:**

The online version contains supplementary material available at 10.1007/s13755-021-00168-2.

## Introduction

Type 2 Diabetes (T2D) is one of the most common severe chronic diseases characterized by progressive complications that include cardiovascular disease, hypertension, retinopathy, kidney disease, and strokes [61, 63]. Pancreas produced insulin controls blood glucose uptake by cells thereby reducing circulating levels; without such glycaemic control circulating sugar levels can remain high for extended periods, resulting in glycation products that have myriad deleterious effects on the body, but notably the vascular system [21]. Type 2 diabetes results from poorly understood processes that cause resistance to insulin stimulation and gradual loss of glycaemic control, which can be accompanied by reduced insulin production. A survey found that 451 million people were globally affected by T2D which will likely increase to 693 million by 2045 [17]. In addition, 85% of T2D patients by 2030 will live in developing countries [40, 63]. However, this disease can generally be prevented or reduced in severity by following healthy lifestyle including a well-balanced diet, exercise and low level psychological stress, however, genetics and environmental factors play a significant role in T2D development [9, 23, 32, 33, 38, 46]. The signs of T2D development and progression include excessive thirst, weight loss, hunger, fatigue, skin problems and slow healing wounds, progressively advancing to life-threatening health issues, as well as significant associations with many other serious comorbidities such as rheumatoid arthritis and Alzheimer’s disease [10, 31, 41, 42, 45]. Given the wide variety of presentation and development of comorbidities in T2D, treatment and care of patients can be greatly improved if the prognostic signs are used to better sub-categorize T2D patients. Machine learning methods are well suited to such categorization tasks and potentially provide useful information to clarify the key symptoms of interest of this disease. The motivation of this work is therefore to develop intelligent T2D detection and categorization models which identifies types of T2D patients and distinguishes them from non-diabetic controls earlier and with greater precision.

**Table 1:** The demographic details of pima Indian diabetes dataset

S/N	Pregnancies	Glucose	BloodPressure	Thickness	Insulin	BMI	DPF	Age
Feature type	Integer	Real	Real	Real	Real	Real	Real	Integer
Unit	Number of times	mg/dL	mm Hg	mm	mu U/ml	kg/m^2^		years
Distinct count	17	136	47	51	186	248	517	52
Unique (%)	2.20%	17.70%	6.10%	6.60%	24.20%	32.30%	67.30%	6.80%
Mean	3.8451	120.89	69.105	20.536	79.799	31.993	0.47188	33.241
Range	0–17	0–199	0–122	0–99	0–846	0–67.1	0.078–2.42	21–81
Zeros (%)	14.50%	0.70%	4.60%	29.60%	48.70%	1.40%	0.00%	0.00%
5-th percentile	0	79	38.7	0	0	21.8	0.14035	21
Q1	1	99	62	0	0	27.3	0.24375	24
Median	3	117	72	23	30.5	32	0.3725	29
Q3	6	140.25	80	32	127.25	36.6	0.62625	41
95-th percentile	10	181	90	44	293	44.395	1.1328	58
Range	17	199	122	99	846	67.1	2.342	60
IQR	5	41.25	18	32	127.25	9.3	0.3825	17
Standard deviation	3.370	31.973	19.356	15.952	115.240	7.884	0.331	11.760
Coef of variation	0.876	0.264	0.280	0.777	1.444	0.246	0.702	0.354
Kurtosis	0.159	0.641	5.180	-0.520	7.214	3.290	5.595	0.643
MAD	2.772	25.182	12.639	13.660	84.505	5.842	0.247	9.586
Skewness	0.902	0.174	-1.844	0.109	2.272	-0.429	1.920	1.130
Sum	2953	92847	53073	15772	61286	24570	362.4	25529
Variance	11.354	1022.2	374.65	254.47	13281	62.16	0.10978	138.3
Memory size	6.1 KB	6.1 KB	6.1 KB	6.1 KB	6.1 KB	6.1 KB	6.1 KB	6.1 KB

However, there are many challenges in designing such kinds of models. T2D is a complex metabolic disorder that contains various types of signs and related comorbid diseases [65]. Identification of major significant features is important for controlling this disease and to utilise effective treatment regimens for affected people. The development and medical costs resulting from T2D are enormous, but there are many poorly defined risk factors. Nevertheless, there has been a great deal of development work in categorizing T2D using various different types of computational methods. In those studies, researchers analyzed T2D patient records to identify more accurate prognostic indicators [25, 54]. However, most of these studies were not able to explore and identify improved working models that have high enough performing features to be usefully employed in the clinic. In this work, we propose an intelligent T2D detection model where different feature selection and classification models have been applied to analyze the T2D dataset to determine out the best classifier. These classification outcomes were then used to explore significant attributes from different perspectives. The contributions of this work are given as follows:

- Newly extended versions of feature selection and classification methods were employed for the analyses of T2D datasets. The proposed model showed greatly improved performance with extended classification models able to recognise T2D better than other existing approaches.
- The classification results of this work are represented with the resampling distribution of summary statistics more accurately. This combination can identify the top performing machine learning model from a range of different viewpoints.
- Finally, non-parametric statistical methods were used to identify the best machine learning model. Then, wireframe contour plots were used to identify the most useful feature subsets with high efficiency.

## Related work

Numerous studies have attempted to predict T2D outcomes using a variety of machine learning techniques [19, 21, 29, 29, 40, 51, 57]. Proposed methods were employed various data preprocessing and machine learning techniques to isolate T2D patients from controls. In data retrieval steps, various techniques such as data cleaning, clustering, sampling, missing value imputation, and outlier detection was used to prepare data for further evaluation. Feature selection methods are also useful to explore the most significant features and reduce computational complexity, including stable outcomes. To analyze T2D detection performance, various widely used classifiers such as K-Nearest Neighbor (KNN), support vector machine (SVM), Naïve Bayes (NB), Artificial Neural Network (ANN), Logistic Regression (LR), Decision Trees (DT), and Random Forest (RF) were implemented. Recently, many ensemble and voting based classification methods have been proposed for such work. [26, 53]. For instance, Kahramani et al. [24] used a hybrid method that mingled ANN and fuzzy neural network (FNN) to predict T2D cases more efficiently. Vaishali et al. [59] used genetic algorithm as feature selection method and applied various classifiers such as multi-objective evolutionary (MOE) Fuzzy, NB, J48 Graft, and Multi Layer Perceptron (MLP) to investigate diabetes dataset. Dagliati et al. [11] considered a data mining pipeline where missing data by means of RF and data balancing strategies were employed, therefore LR with stepwise feature selection and different classifiers were used in that analysis. In addition, Maniruzzaman et al. [30] used a range of feature selection methods, including principal component analysis (PCA), Analysis of Variance (ANOVA), mutual information (MI), LR, and RF) in the PIDD analysis to explore various subsets and then classify them with various classifiers. Also, Wei et al. [64] used deep neural network (DNN) in preprocessed PIDD (i.e., applying scaling, normalization, imputation and dimensionality reduction method) and showed highest 77.86% accuracy. Thus, Battineni et al. [6] employed KNN to impute missing records as well as NB, J48, LR, and RF were implemented for investigating T2D datasets. Wang et al. [63] proposed a method named Prediction algorithm for the classification of T2D on imbalanced data with Missing values (DMP_MI) where NB compensated this missing records. The adaptive synthetic sampling method (ADASYN) was then used to balance this dataset and applied RF to achieve a classification result. Hasan et al. [17] proposed a machine learning methodology where they implemented PCA, Independent Component Analysis (ICA) and Correlation-based Feature Selection (CFS) for feature selection and employed KNN, DT, RF, AdaBoost, NB, XGBoost, and MLP as classification techniques. Tripathi and Kumar [55] used random oversampling (ROS), normalization, and several classifiers like Linear Discriminant Analysis (LDA), KNN, SVM, and RF were used to investigate their primary diabetes dataset for machine learning purposes. Ismail et al. [22] provided a taxonomy of significant factors where different machine learning algorithms were used with or without feature selection processing. In addition, Ramesh et al. [44] implemented multivariate imputation by chained equations (MICE) method for handling missing values of primary diabetes dataset. Subsequently three feature selections (chi-squared test, extremely randomized trees, and least absolute shrinkage and selection operator (LASSO)) and some classifiers such as KNN, LR, Gaussian NB, and SVM were used to investigate this dataset. Meanwhile, Banerjee and Satyanarayana [4] created an ensemble learning method called SDS where DT, stochastic gradient boosting (SGD), and gradient boosting classifier (GBC) are incorporated to find its highest results. Some deep learning approach had been applied into diabetes dataset to get more suitable results for detecting diabetes [27, 34]. For example, Gupta et al. [16] used deep learning (DL) and quantum machine learning (QML) to detect diabetes where DL outperformed related QML algorithms.

## Materials and methods

**Fig. 1:**
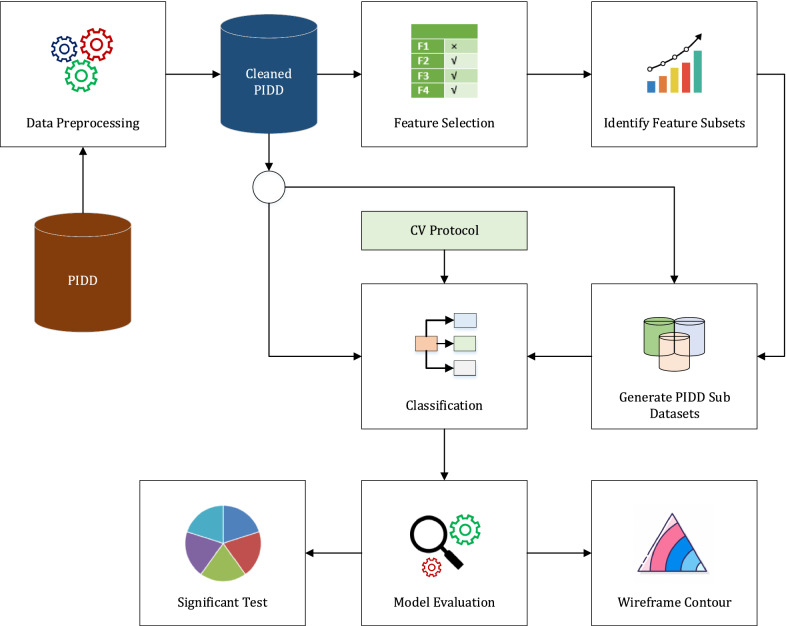
Proposed methodology

Several steps were considered to analyze T2D dataset and its feature subsets by implementing a number of high performing classifiers which are given as follows (see Fig. 1).

### Machine learning based diabetes detection model

- *Data Description and Preprocessing* In this work, we employed a widely used dataset, PIDD obtained from the publicly available Kaggle ML Repository, provided by the National Institutes of Diabetes, Digestive and Kidney Diseases [37]. All of the subjects were females over 21 years old of Pima Indian indigenous heritage from a population near Phoenix, Arizona, USA. It provides 768 patient records with 9 features where 268 patients (34.9%) had T2D and 500 patients (65.1%) were non-diabetic (see details in Table 1). PIDD contains personal health data from medical examination and does not have missing values, but required some cleaning and removal of unwanted instances from the dataset.
- *Feature Selection Approach* Feature selection methods are used to interpret and reduce variation and computational cost of processing training datasets. After performing preprocessing steps, different feature subsets were identified from PIDD using a number of feature selection methods such as information gain attribute evaluation (IGAE), gain ratio attribute evaluation (GRAE), gini indexing attribute evaluation (GIAE), analysis of variance (ANOVA), chi-square (${\tilde{\chi }}^2$) test, extension of relief (reliefF) attribute evaluation (RFAE), correlation based feature selection subset evaluation (CFSSE). fast correlation based feature selection (FCFS), and filter subset evaluator (FSE). These methods have been widely used in many previous machine learning studies [20, 30]. After these steps, these feature subsets were used to generate sub datasets from PIDD.
- *Classification* Numerous classification models (i.e., almost 184 classifiers) were implemented to scrutinize primary and its sub datasets. However, some of these required long computation times and were not supported on these datasets, therefore, we discarded them. Finally, ten classifiers like boosted generalized additive model (GAMBoost), regularized LR (RLR), penalized multinomial regression (PMR), Bayesian generalized linear model (BGLM), penalized LR (PLR), generalized linear model (GLM), sparse distance weighted discrimination (SDWD), generalized boosted regression modeling (GBM), generalized additive model using LOESS (GAMLOESS) and NB were employed in the PIDD data along with its sub-datasets. In this work, we considered cross validation (CV) protocol for each classifier to analyze T2D data. In this case, the re-sampling technique were used for the machine learning models by dividing instances into k groups (randomly constructed of approximately equal size) where the specific (k) fold was treated as a validation set, along with remaining k-1 folds. Different evaluation metrics such as accuracy, kappa-statistics, AUROC, sensitivity, specificity, and logloss were used to investigate the performance of different classifiers.
- *Investigating Derived Results* The classification outcomes were analyzed to identify the best models (see details in “Experimental results” section). Furthermore, non-parametric Friedman Tests [51], along with Iman-Davenports ($F_{ID}$) adjustment was implemented into the generated results to verify the predictive performance of individual classifiers as well as identify the best performing classifier. To explore the best feature subsets, we investigated the optimum combination of datasets and classification results to identify the significant feature subsets where different classifiers had shown good performance.

However, a brief description of the various feature selection and classification methods are provided as follows:

#### Feature selection approach

The general description of individual feature selection methods is given as follows.

- *Information Gain Attribute Evaluation (IGAE)* compares the entropy of the dataset before and after transformation [50]. It is preferable to identify significant attributes from a large number of features. Suppose $S_x$ is the set of training samples where information gain (*IG*) is determined for a random variable $x_i$ using following equation:
  1 $$\begin{aligned} IG(S_x,x_{i})=H(S_{x})-\sum _{v} \frac{ | S_{x=v} | }{ | S_{x} | } H(S_{x_i}) \end{aligned}$$
- *Gain Ratio Attribute Evaluation (GRAE)* is the extension of IG that lessens its biasness using intrinsic information (i.e., entropy of data distribution in branches) [39]. Therefore, the gain ratio of attribute *A* is shown as follow:
  2 $$\begin{aligned} \text {GR}(A) = \frac{\text {{IG}}(A)}{{\text {Intr}_\text {info}}(A)} \end{aligned}$$
  where $\text {Intr}_\text {info}$ is denoted as Intrinsic Information.
- *Gini Indexing Attribute Evaluation (GIAE)* was used to select most splitting features from nodes [35]. However, bias remains in the unbalanced datasets that contain a large numbert of attributes. Besides this, Gini indexes provide low values for stubby frequent attributes and high values for top frequent attributes. However, these values are relatively lower for specific attributes of larger classes.
- *Analysis of Variance (ANOVA)* is a parametric statistical hypothesis test where the means of two or more samples are checked and ensured their same distribution or not [30]. It uses an F-test to determine the significant difference between samples. Therefore, it contrasts between-groups variability to within the group variability using F-distribution.
- *Chi-Square* (${\tilde{\chi }}^2$) **Test** compares the independence of different variables. It uses $\chi ^2$ statistics to measure the strength of the relationship between independent features [60]. In this method, higher $\chi ^2$ values of features are more dependent on the response [28]. Hence, this method is calculated using following equations:
  3 $$\begin{aligned} {\tilde{\chi }}^2= \sum _{i=1}^r \sum _{j=1}^c \frac{( {O_{i,j}- E_{i,j})}^{2}}{E_{i,j}} \end{aligned}$$
- *Extension of Relief Attribute Evaluation (RF-AE)* is a filter based method that is notably sensitive regarding feature interaction. Relief score ($R_x$) determines the value of each attribute and ranks them for feature selection. This score is calculated based on the selection of attribute value differences between nearest neighbor instance pair of different and same classes [58]. It defines as follows:
  4 $$\begin{aligned} R_x=P({\text {diff}} X|{\text {diff}} class)-P({\text {diff}} X |{\text {same}} class) \end{aligned}$$
  In this case, if a attribute value difference is found for the same classes, then the relief score is decreased. Otherwise, this score is increased.
- *Correlation based Feature Selection (CFS)* measures the importance of individual features by computing inter-correlation values among them. In this method, highly correlated and irrelevant features are avoided [7] to identify the most significant features from the dataset. Also, different methods like best first search (BFS), evolutionary search (ES), reranking search (RS), scatter search (SS) and other related methods are employed with CFS to explore significant features.
- *Fast Correlation based Feature Selection (FC-FS)* [3] is a multivariate method that has symmetrical uncertainty to determine feature dependencies and find the corresponding subset using backward selection procedure.
- *Filter Subset Evaluation (FSE)* is employed with an arbitrary filter (SpreadSubsampler) when different instances are passed through this filter and identified significant features.

#### Classification approaches

1. *Boosted Generalized Additive Model (GAMBoost)* is transformed each predictor variables and generated a weighted sum of them in a nonlinear way [56]. Each predicting component is fitted with the residuals to minimize prediction cost of this model.
2. *Regularized Logistic Regression (RLR)* contains one or more independent variables [18, 66] that represents hypothetical outcomes considering logistic or sigmoid function using regularization term. It is also prone over fitting if there are a large number of features. Let, $x={x_1,x_2,\ldots \ldots ,x_n}$ independent variables and $\theta ={\theta _1,\theta _2,\ldots \ldots ,\theta _n}$ parameters are considered where the expected result $h_{\theta }(x)$ is:
  5 $$\begin{aligned} h_{\theta }(x)=\frac{1}{1+e^{\theta ^{T}x}} \end{aligned}$$
  where $1 \le h_{\theta }(x) \le 1$. So, the cost function $MSE(\theta )$ of LR can be expressed as:
  6 $$\begin{aligned}&E_{\theta }(i)= y(i)log(h_{\theta }(x(i)) \end{aligned}$$
  7 $$\begin{aligned}&F_{\theta }(i)= (1-y(i))log(1-h_{\theta }(x(i))) \end{aligned}$$
  8 $$\begin{aligned}&MSE(\theta )=- \frac{1}{m} \sum _{i=1}^{m} E_{\theta }(i) + F_{\theta }(i) \end{aligned}$$
  The cost function is updated by the penalized high values of a parameter called regularization term $\frac{ \lambda }{2m} \sum _{j=1}^n \theta ^2$ (i.e., $\lambda$ is the regularization factor) that is also expressed as:
  9 $$\begin{aligned} J(\theta )= MSE(\theta ) +\frac{\lambda }{2m}\sum _{j=1}^n \theta ^2 \end{aligned}$$
  Regularization in LR is useful to generalize better on unseen data and prevent overfitting of training data.
3. *Penalized Multinominal Regression (PMR)* is a mixture logit model that initiates with a penalty to eliminate the infinite number of components from the maximum likelihood estimators [5]. Ridge regression is a simple form of penalized regression which handles multicollinearity of regressors (i.e., following linear regression). This penalization approach helped to avoid an overfitting problem.
4. *Bayesian Generalized Linear Model (BGLM)* is a generalization of linear regression model where statistical analysis is happened in the context of Bayesian inference. In this case, Bayes estimation remains consistent with true value by its prior support. This approach is used to estimate linear model coefficients with external information. Moreover, the complexity of BGLM gives uncertainty which leads to the natural regularization. Hence, LASSO and other regularized estimators are represented as Bayesian estimators for a particular prior [14].
5. *Penalized Logistic Regression (PLR)* creates a regression model with a large number of variables using the logistic or sigmoid function. Three regression models, such as ridge, LASSO and elastic regression are mingled which shrinks low-contributing factors towards zero [8]. Ridge regression follows L2 regularization where the penalty term $\frac{ \lambda }{2m} \sum _{j=1}^n \theta ^2$ is used to the cost function.
  10 $$\begin{aligned} J(\theta )= MSE(\theta ) + \frac{ \lambda }{2m} \sum _{j=1}^n \theta ^2 \end{aligned}$$
  Besides, L1 regularization is considered by LASSO regression where following penalty term $\frac{ \lambda }{2m} \sum _{j=1}^n |\theta |$ is used.
  11 $$\begin{aligned} J(\theta )= MSE(\theta )+ \frac{ \lambda }{2m} \sum _{j=1}^n |\theta | \end{aligned}$$
  Elastic net is a combination of L2 and L1 regularization penalties to define cost function.
  12 $$\begin{aligned} J(\theta )= MSE(\theta ) + \frac{ \lambda }{2m} \big ( \frac{1-\alpha }{2} \sum _{j=1}^n |\theta | + \alpha \sum _{j=1}^n \theta ^2 \big ) \end{aligned}$$
  Like the other regression models, it minimizes cost function $J(\theta )$ and maximize its outcomes.
6. *Generalized Linear Model (GLM)* is a induction of linear regression which gathers systematic and random components in a statistical models. Suppose, a set of independent variables $x_0,x_1,\ldots ..,x_n$ with some coefficients $\theta ={\theta _0,\theta _1\ldots \ldots ..,\theta _n}$ is used to build following hypothesis [18]:
  13 $$\begin{aligned} h_{\theta }(x)=\theta ^{T}x= \theta _0+\theta _1x_1+\theta _2x_2+\ldots \ldots ..+\theta _nx_n \end{aligned}$$
  Besides, the cost function of GLM is represented as:
  14 $$\begin{aligned} J(\theta )=- \frac{1}{2m} \sum _{i=1}^ m(h_{\theta }(x)-y)^2 \end{aligned}$$
  After generating the cost function $J(\theta )$, minimizing is needed to get more accurate results in data analysis.
7. *Sparse Distance Weighted Discrimination (SD-WD)* represents $l_1$ Distance Weighted Discrimination (DWD) (i.e., by following $l_1$ SVM) by replacing $l_2$ DWD in order to achieve sparsity and show its lost and penalty. If $l_2$ norm penalty is used, the performance of all high dimensional variables is very poor [62]. Therefore, Zhu et al. [67] proposed the $l_1$-norm SVM to fix this problem. It provides efficient computational performance for extensive numerical experiment.
8. *Generalized Boosted Regression Model (GBM)* is the combination of various decision trees and boosting methods where these decision trees are fitted repeatedly to improve the performance of the model. In this case, a random data subset is selected from each new tree using a boosting method whereby the first tree is fitted and next tree is taken based on the residuals. Thus, this model tries to improve accuracy at every step. It explores the combination of related parameters which determines minimum error for predictions with at least 1000 trees (i.e. following sufficient shrinkage rates) [12, 13].
9. *Generalized Additive Model using LOESS (G-AMLOESS)* utilizes linear predictor along with locally weighted regression (LOESS) to fit on smooth 2D in the 3D surfaces. Let *Y* be a univariate response variable where $x_i$ is defined with various continuous, ordinal and normal predictors. Furthermore, different distributions such as normal, binomial or poisson distributions as well as link functions like identity and log functions are used to get the expected value of *Y*.
  15 $$\begin{aligned} g(\mu ) = \beta _0 + f_1(x_1) + f_2(x_2) + \ldots + f_k(x_k) \end{aligned}$$
10. *Naïve Bayes (NB)* is a probabilistic classifier which is based on Bayes theorem with the strong independent assumption between the features. It is particularly useful for large datasets. In addition, the presence of particular features are not related with any others which is manipulated by the following condition [15]:
  16 $$\begin{aligned} P(c|X)= \frac{P(X|c)P(c)}{P(X)} \end{aligned}$$
  where *P*(*c*|*X*) is called posterior probability of class for given predictor. Then, $P(X|c)=P(x_1|c) \times P(x_2|c) \times P(x_3|c) \times \ldots . \times P(x_n|c) \times P(c)$ , *P*(*c*|*x*), *P*(*c*), *P*(*x*|*c*) is defined as likelihood. Besides, *P*(*c*) and *P*(*X*) are represented as prior probability and marginal respectively.

### Performance measures

A confusion matrix describes the performance of a classification model using the number of false-positive (*FP*), false negative (*FN*), true positive (*TP*) and true negative (*TN*) values. Several evaluation metrics such as accuracy, kappa statistics, AUROC, sensitivity, specificity, and logarithmic loss are used to justify the outcomes of different classifiers [47, 48, 50]. Therefore, a brief description of them is given as follows:

#### Evaluation metrics

- *Accuracy* indicates the ratio between correct and overall number of predictions which is provided as follows:
  17 $$\begin{aligned} Accuracy =\left( \frac{TP+TN}{TP+FN+FP+TN}\right) \end{aligned}$$
- *Kappa Statistics* defines the inter rater agreement of observed and expected accuracy for qualitative features.
  18 $$\begin{aligned} K_{p}=1-\frac{1-p_{o}}{1-p_{e}} \end{aligned}$$
- *Average area under receiver operating characteristic (AUROC)* is calculated from true positive rate/sensitivity and (1-false positive rate)/specificity for all possible orderings. Let, $t_n$ and $t_{n-1}$ are considered as the time observation of the concentration $C_n$ and $C_{n-1}$ respectively. Therefore, AUROC can be defined as:
  19 $$\begin{aligned} \mathrm {[AUROC]}_{n-1}^{n}=\frac{\mathrm {C}_{n-1}+\mathrm {C}_{n}}{2} \cdot \left( \mathrm {t}_{n}-\mathrm {t}_{n-1}\right) \end{aligned}$$
- *Sensitivity* represents the proportion of correctly classified positive and all positive instances.
  20 $$\begin{aligned} \text{ Sensitivity }=\left( \frac{TP}{TP+FN}\right) \end{aligned}$$
- *Specificity* determines from the proportion of correctly classified negative and all the negative instances.
  21 $$\begin{aligned} \text{ Specificity }=1-\left( \frac{FP}{FP+TN}\right) \end{aligned}$$
- *Logarithmic loss (Logloss)* assesses the performance of individual classifiers by following equation
  22 $$\begin{aligned} L_{g}=\frac{-\sum _{y=1}^{j} \sum _{x=1}^{n} f(x, y) \log (p(x, y))}{n} \end{aligned}$$

### Friedman test

Friedman test is a non-parametric statistical method which considers *p* with $k-1$ degrees of freedom under the null hypothesis and their outcomes do not rapidly change in all machine learning approaches. $P_i$ is indicated as the average rank over *N* training sets of a classifier. If the null hypothesis is not accepted, the best classifier is assessed pairwise with each standard algorithm using several post-hoc tests, including Bonferroni, Holm and Holland. Thus, Iman-Davenport and Friedman statistics are defined as:

23 $$\begin{aligned}&F_{ID}=\frac{(N-1) X_{F}^{2}}{N(K-1)-X_{F}^{2}} \end{aligned}$$

24 $$\begin{aligned}&X_{F}^{2}=\frac{12N}{k(k+1)} \sum _{i=1}^{k}\left( P_{i}^{2}-\frac{k(k+1)}{4}\right) ^{2} \end{aligned}$$

## Experimental results

### Experimental settings

In this work, we implemented the following feature selection methods (FSM) in the PIDD and generated various feature subsets (i.e., FS1, FS2, FS3, FS4, FS5, and FS6) using Orange v3.29.1 and Waikato Environment for Knowledge Analysis (WEKA 3.8.5). We conjugated various searching methods such as BFS, ES, RS, and SS with different attribute selector of WEKA. In this case, we selected the top 5 ranked attributes for each method using Orange software. Table 2 shows the list of feature subsets sequentially. This process resulted in different sub-datasets (DS1, DS2, DS3, DS4, DS5, and DS6) of PIDD formulated based on the feature subsets. Various classifiers (almost 184) were then employed to analyze these datasets using caret package in R (3.5.1). However, proposed top ten stable classifiers were identified to evaluate automatic diabetes detection process more accurately. To visualize the resampling distribution of summary results (i.e. minimum, mean, median and maximum findings), we utilized the matplotlib library using python in the Google Colaboratory platform. Finally, non-parametric Friedman Test was applied to derived classification results to explore significant classification model by assessing overall results using Knowledge Extraction based on Evolutionary Learning (KEEL GPLv3).

**Table 2:** Formulation of Various Feature Subsets

FS	FST	Tool	SM/TS	Features
FS1	IGAE	Orange	Top 5	Glucose, Age, BMI, Insulin, and
	GRAE	Orange	Top 5	Pregnancies
FS2	GIAE	Orange	Top 5	Glucose, BMI
	ANOVA	Orange	Top 5	Age, DPF, and
	X2 test	Orange	Top 5	Pregnancies
FS3	RFAE	Weka	Ranker, Top 5	Glucose, Age, Pregnancies, Thickness, and BMI
FS4	FCFS	Orange	Top 5	Glucose, Age, BMI, DPF, and Insulin
FS5	CFS	Weka	BFS, ES, RS, SS	Glucose, BMI, DPF, and Age
FS6	FSE	Weka	BFS	Glucose, BMI, and Age

### Investigating the classification performance of diabetes detection

To scrutinize PIDD and its sub-datasets, various classifier models including GAMBoost, RLR, PMR, BGLM, PLR, GLM, SDWD, GBM, GAMLOESS and NB were considered. In this case, we identified the best classifiers to determine the accurate results along with significant features for detecting T2D. Then, the experimental outcomes of them were justified. In this work, the summary statistical results are organized by resampling distribution. The details of these findings are shown in Supplementary Table 1–6, respectively.

The accuracy of these classifiers are given in Supplementary Table 1. In this work, GAMLOESS provided minimum highest accuracy (71.05%) for DS4. However, many classifiers gave the top median accuracy (77.92%) for different datasets. Consequently, RLR, BGLM, PLR, and SDWD showed the best median accuracy for PIDD and SDWD provided the highest median accuracy for DS2. Also, GAMBoost, RLR, PMR, BGLM, PLR, and GLM for DS5 and GAMLOESS for DS6 produced similar results. Thus, GAMBoost presented the best mean accuracy of 77.73% for DS5. Besides this GBM gave the greatest maximum accuracy of 90.91% for DS4.

Kappa statistics for individual classifiers are shown in Supplementary Table 2. GAMLOESS determined the supreme minimum kappa of 31.42% for DS4. Besides, GAMBoost provided the best median kappa (49.87%) for DS5. On the other hand, NB showed the top mean kappa of 48.97% for DS2. Finally, GBM exhibited the utmost maximum kappa of 78.77% for DS4.

The AUROC values of different classifiers are given in Supplementary Table 3. GAMLOESS generated the highest minimum (76.92%), median (85.36%) AUROC for FS5 and FS6 respectively. NB provided the supreme mean AUROC of 84.84% for DS5. For DS3, GAMLOESS showed the best maximum AUROC, of 95.26%.

The sensitivity of the following classifiers is given in Supplementary Table 4. SDWD gave the highest minimum (96%), median (100%), mean (99.2%) and maximum (100%) sensitivity for DS6 (see Supplementary Table 4). In addition, SDWD and GBM gave the theoretical maximum sensitivity (100%) for DS5 and DS2 respectively.

In addition, NB showed the highest minimum (44.44%) and median (62.96%) specificity for DS2. Again, this classifier provided the highest minimum (44.44%), median (62.96%) and mean (62.23%) specificity for DS3 respectively. Besides this, NB showed the top median specificity (62.96%) for DS6. However, GBM manipulated the utmost maximum specificity (85.19%) for DS6.

When the experimental result with logloss was analyzed (see Supplementary Table 6), NB gave the lowest minimum logloss (30.98%) for DS4. GAMLOESS gave the lowest median logloss of 45.58% for DS6. In contrast, GAMBoost provided the shallow mean (46.43%) for DS5. Afterwards, this classifier presented the stubby maximum logloss of 56.83% for DS4.

**Fig. 2:**
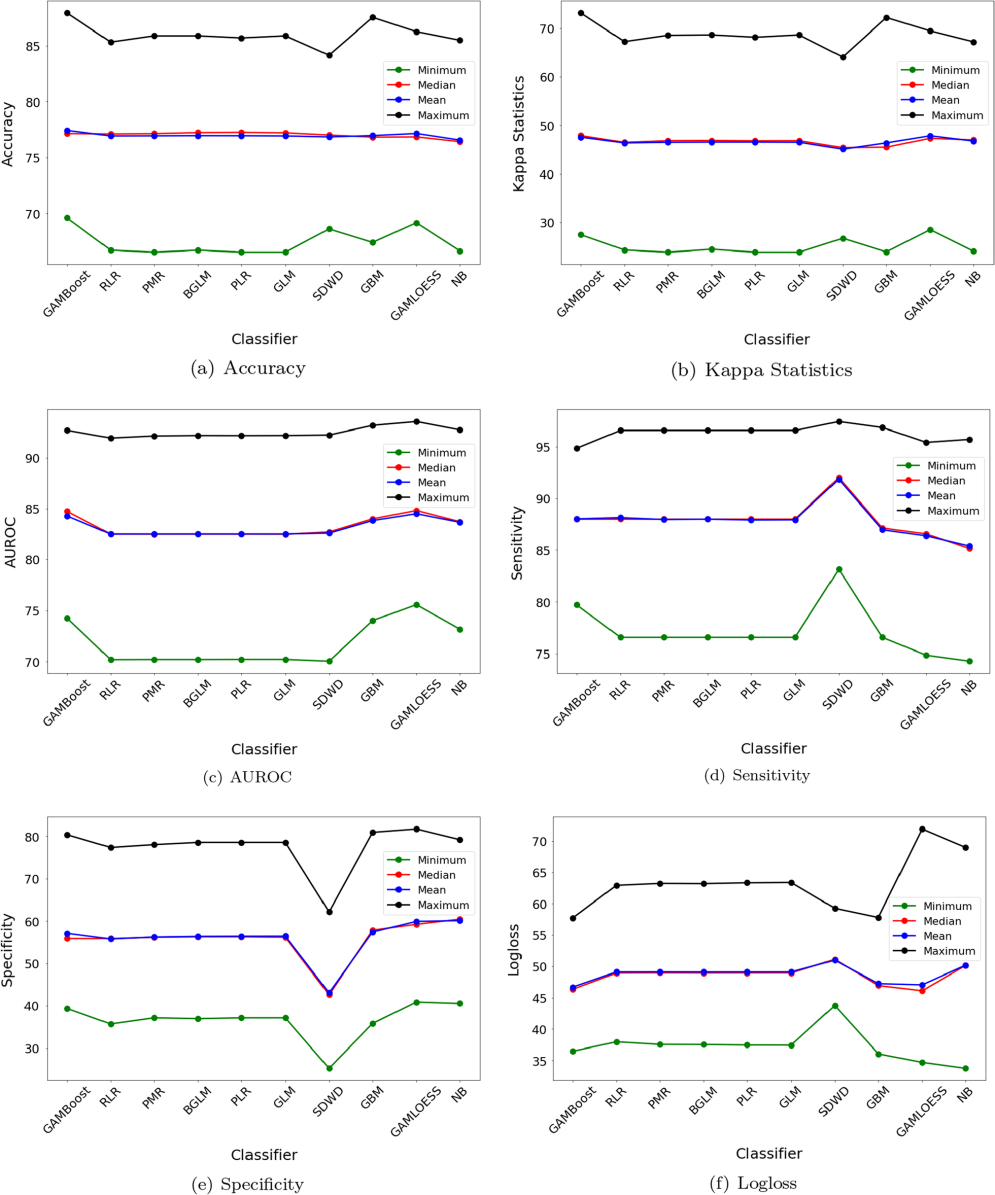
Average (Minimum, Median, Mean, and Maximum) Results of Different Classifiers

**Fig. 3:**
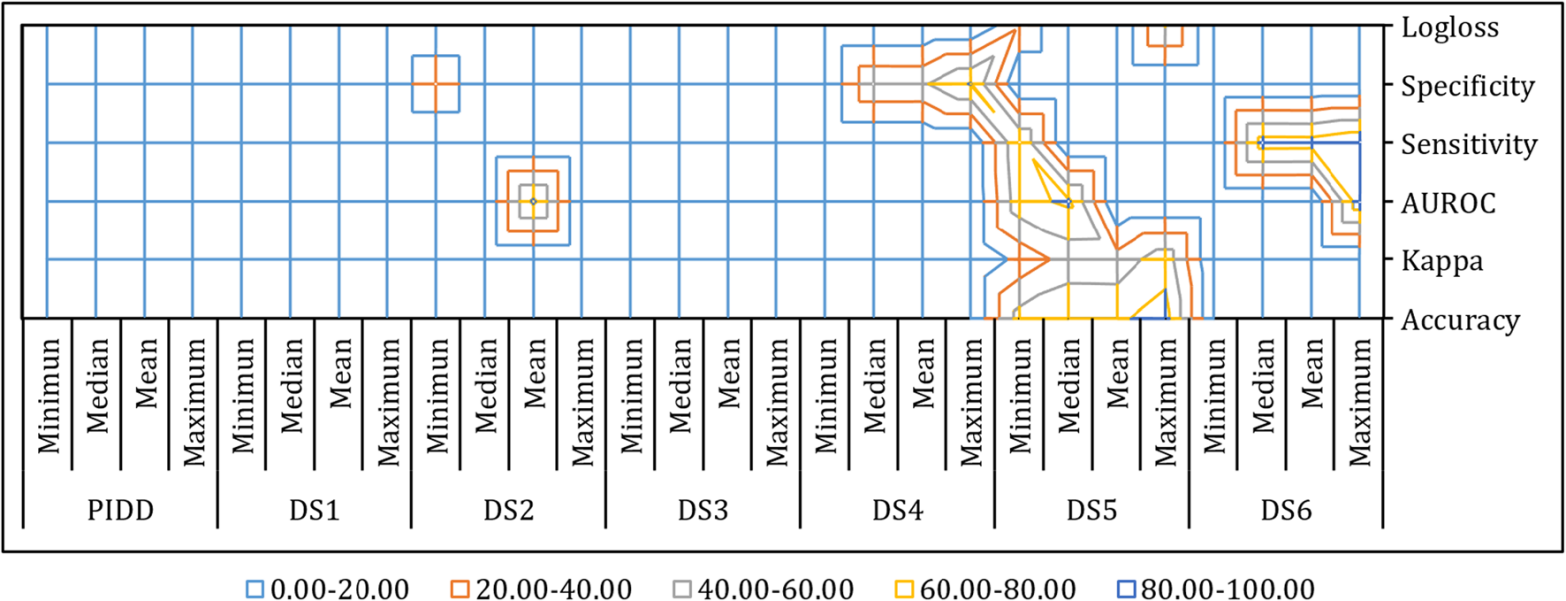
Wireframe Contour of Average Best Classification Results for Individual Datasets

The average minimum, median, mean and maximum accuracy, kappa statistics, sensitivity, AUROC, specificity and logloss are visualized at Fig. 2. The average best classification results for different datasets are illustrated with wireframe contours maps in Fig. 3.

**Table 3:** Classifiers Ranking & Adjusted P-values using Post Hoc Methods (Friedman) based on Average Findings

i	Classifier	Ranking	$z=\frac{R_0-R_i}{E}$	Unadjusted p	$p_{Bonf}$	$p_{Holm}$	$p_{Hochberg}$
1	GAMLOESS	3.00					
2	GAMBoost	3.17	0.10	0.9240	8.3164	0.9240	0.9240
3	GBM	5.00	1.14	0.2526	2.2730	0.5458	0.5051
4	SDWD	5.33	1.33	0.1819	1.6373	0.5458	0.5051
5	BGLM	5.67	1.53	0.1271	1.1441	0.5167	0.5051
6	GLM	5.92	1.67	0.0952	0.8568	0.5167	0.4760
7	NB	6.00	1.72	0.0861	0.7751	0.5167	0.4760
8	PLR	6.67	2.10	0.0359	0.3235	0.2516	0.2516
9	PMR	6.92	2.24	0.0251	0.2254	0.2004	0.2004
10	RLR	7.33	2.48	0.0132	0.1186	0.1186	0.1186

## Discussion

### Comparing classification performances and identifying significant feature subsets

In this study, we analyzed PIDD and its sub-datasets using various classifiers to identify the best classifier based on experiment results. In all cases giving the best results for individual classifiers, GBM gave the highest maximum accuracy (90.91%) and maximum kappa statistics (78.77%) for DS4 respectively. Also, this classifier provided the best specificity for DS6. Then, SDWD showed the top sensitivity (100%) for DS5 and GAMLOESS gave the maximum AUROC of 95.26% for DS3. However, GAMBoost obtained the lowest logloss for DS4 respectively. However, the overall best classifier were not identified from this analysis. The average outcomes (i.e., accuracy, kappa statistics, AUROC, sensitivity, specificity and logloss) of individual classifiers were used to explore the best classification approach (see Fig. 2). Among all classifiers, GAMBoost and GAMLOESS provided the best outcomes in this analysis. That is to say that, GAMBoost gave a better performance than GAMLOESS for accuracy, sensitivity (see Fig. 2a, c) while, GAMLOESS showed better results for AUROC and specificity (see Fig. 2d, e). GAMBoost and GAMLOESS gave comparable results for kappa statistics and logloss. However, the performance of other classifiers was not consistent for different evaluation metrics; these included GAMBoost and GAMLOESS. Therefore, we again averaged minimum, median, mean and maximum results of different classifiers and used Friedman test to conduct non-parametric statistical analysis among them (see Table 3). This showed that GAMLOESS as the best ranked classifier (#1) to correctly classify diabetes outcomes, while GAMBoost was the second best (#2) ranked algorithm.

In the 2D wireframe contour graph noted above, the average highest classification outcomes are illustrated only for those datasets where classifiers provide the best average outcomes. This surface chart is helpful to extract the optimum combination of datasets for minimum, median, mean and maximum outcomes. Shown in Fig. 3 is the optimum combination of average highest performance found for DS5. The other amalgamation of surfaces are visualized for DS6, DS4 and DS2, respectively. As a result, Glucose levels, FS5 is found to be the most consistent feature subset which produces frequent outcomes. In addition, FS6, FS4 and FS2 can be also considered as the significant feature subsets where numerous classifiers can generate good and consistent results. Furthermore, we have provided the average highest classification outcomes for different datasets in Supplementary Table 7.

### Comparing results with previous studies

A number of studies have previously been performed on this PIDD data but their outcomes were not useful in some respects. Therefore, we proposed an intelligent computing diabetes detection model which fixes some of these issues to provide more suitable outcomes. Most of the machine learning related PIDD studies were used different kinds of general data processing approaches (i.e.,identifying/removing/replacing missing words and deleting wrong values) and advanced approaches such as data transformation [1, 2, 27], outlier detection [43], removal or replacement with mean or median values. [30, 49]. In real-time data analysis, most of a dataset contains significant numbers of outliers and extreme values. In this study, the general procedures of data cleaning are followed to pre-process and generate better results. In previous studies, many researchers had used unsupervised clustering methods to gather more similar instances into homogeneous group [51, 55]. Nevertheless, numerous similar instances of clusters were not matched with regular classes, so need to remove them from analysis [35, 65]. In our proposed model, we avoided more pre-processing approaches to keep practical characteristics of PIDD.

In the current study, we applied different types of standard classifiers and extended these to use on the PIDD and its feature subsets, which did not use many state-of-art techniques [1, 30, 35, 51]. Many previous studies researchers had not employed about feature subsets evaluation [36, 52, 65]. However, in this work, different standard and augmented classifiers were used to investigate their performance based on resampling distribution (i.e., minimum, median, mean, and maximum) of summary statistics. Therefore, the performance of individual classifiers was scrutinized more carefully. Also, we used non parametric Friedman testing to make a priority list of individual classifier. It should also be noted that the wireframe contour plot efficiently depicted the most significant feature subsets which were not identified in previous studies.

In this work, the performance of individual classifiers were not assessed with more T2D datasets. We did not fully compare the performance of the existing model with extended classifiers because the evaluation metrics of them are not same.

## Conclusion and future work

In this work, we investigated the PIDD T2D dataset using various statistical, machine learning and visualization techniques to determine the ranking of classifiers and feature subsets. We found that GAMLOESS was the top ranked classifier and FS5 was the most significant feature subset for achieving the best classifications and analyzing this disease. Note that this T2D dataset which we used, is not very large. In future, the performance of this model will be inspected using multiple diabetes datasets and explored with high performing machine learning models for various crucial features which will enable us better classify this disorder. This work, therefore, has potentially significant clinical importance and the study outcomes method developed will help physicians and researchers to predict T2D more reliably.

## Supplementary Information

Below is the link to the electronic supplementary material.

Supplementary file1 (PDF 41 kb)
