# Supplementary material for: Machine learning models for classification and identification of significant attributes to detect type 2 diabetes
Source: Health Inf Sci Syst. 2022 Feb 9;10(1):2. doi: 10.1007/s13755-021-00168-2 (PMC8828812; doi:10.1007/s13755-021-00168-2)
Supplement: Supplementary file 1 — Supplementary file1 (PDF 41 kb) [file 13755_2021_168_MOESM1_ESM.pdf]

Table 1: Accuracy of Different Classifiers

		GAMBoost	RLR	PMR	BGLM	PLR	GLM	SDWD	GBM	GAMLOESS	NB
PIDD	Minimum	69.74	67.11	67.11	67.11	67.11	67.11	69.74	68.42	69.74	67.11
	Median	76.62	**77.92**	77.63	**77.92**	**77.92**	77.63	**77.92**	76.47	76.62	75.32
	Mean	77.47	77.55	77.44	77.50	77.57	77.44	77.14	76.67	76.87	75.62
	Maximum	88.31	84.42	84.42	84.42	84.42	84.42	84.21	85.53	85.71	84.42
DS1	Minimum	69.74	67.11	67.11	67.11	67.11	67.11	67.11	65.79	68.42	64.47
	Median	77.13	76.47	76.47	76.47	76.47	76.47	76.47	76.62	77.27	76.62
	Mean	77.03	76.33	76.51	76.51	76.51	76.51	76.38	77.11	77.18	75.59
	Maximum	87.01	84.42	85.71	85.71	85.71	85.71	83.12	88.31	85.71	84.42
DS2	Minimum	69.74	65.79	65.79	65.79	65.79	65.79	69.74	68.42	68.42	65.79
	Median	76.62	77.13	77.13	77.13	77.13	77.13	**77.92**	76.62	76.62	76.62
	Mean	77.60	76.95	76.95	76.92	76.95	76.95	77.16	76.72	77.06	77.44
	Maximum	88.31	83.12	85.71	85.71	84.42	85.71	84.21	84.42	84.42	85.71
DS3	Minimum	69.74	65.79	65.79	65.79	65.79	65.79	67.11	67.11	67.11	66.23
	Median	76.62	77.13	77.13	77.13	77.13	77.13	75.82	77.13	76.47	76.62
	Mean	77.08	76.48	76.51	76.48	76.51	76.51	76.38	77.21	76.77	76.27
	Maximum	87.01	85.71	85.71	85.71	85.71	85.71	83.12	87.01	87.01	84.42
DS4	Minimum	69.74	68.42	67.11	68.42	67.11	67.11	69.74	66.23	**71.05**	65.79
	Median	77.27	76.62	77.78	77.63	77.78	77.78	77.27	76.62	76.47	76.62
	Mean	77.55	77.27	77.29	77.37	77.29	77.29	77.08	76.83	77.19	76.93
	Maximum	88.31	85.71	85.71	85.71	85.71	85.71	84.42	**90.91**	88.31	86.84
DS5	Minimum	68.83	67.11	67.11	67.11	67.11	67.11	69.74	68.83	69.74	68.83
	Median	**77.92**	**77.92**	**77.92**	**77.92**	**77.92**	**77.92**	77.13	76.62	76.62	76.62
	Mean	**77.73**	77.32	77.29	77.32	77.29	77.29	77.03	76.80	77.42	77.19
	Maximum	89.61	88.31	88.31	88.31	88.31	88.31	84.42	88.31	85.71	85.71
DS6	Minimum	69.74	65.79	65.79	65.79	65.79	65.79	67.11	67.11	69.74	68.42
	Median	**77.92**	76.62	75.97	76.47	76.47	76.47	76.62	77.78	**77.92**	76.62
	Mean	77.60	76.69	76.66	76.66	76.59	76.61	76.82	77.47	77.63	76.98
	Maximum	87.01	85.71	85.71	85.71	85.71	85.71	85.71	88.31	87.01	87.01

Table 2: Kappa Statistics of Different Classifiers

		GAMBoost	RLR	PMR	BGLM	PLR	GLM	SDWD	GBM	GAMLOESS	NB
PIDD	Minimum	28.13	24.84	24.84	24.84	24.84	24.84	28.75	24.80	28.13	24.84
	Median	46.86	48.17	48.28	48.67	48.67	48.28	46.51	43.66	46.86	44.01
	Mean	47.34	47.84	47.65	47.73	47.96	47.65	45.27	45.35	47.29	44.57
	Maximum	73.66	64.57	64.57	64.57	64.57	64.57	64.26	68.14	68.89	64.57
DS1	Minimum	26.68	25.17	25.17	26.24	25.17	25.17	24.84	22.57	29.85	20.34
	Median	47.17	45.20	45.65	45.20	45.65	45.65	44.65	45.42	48.08	45.85
	Mean	46.74	45.19	45.65	45.60	45.65	45.65	44.26	46.66	47.85	44.15
	Maximum	70.99	65.78	68.89	68.89	68.89	68.89	61.95	74.11	67.81	63.93
DS2	Minimum	28.13	22.57	22.57	22.57	22.57	22.57	28.75	27.16	25.73	22.57
	Median	47.14	45.71	46.68	46.68	46.87	46.68	46.51	44.36	47.32	49.44
	Mean	47.66	46.03	46.36	46.28	46.37	46.36	45.34	45.50	47.69	**48.97**
	Maximum	73.66	61.95	67.81	67.81	64.57	67.81	64.26	65.18	65.18	67.81
DS3	Minimum	26.68	24.00	24.00	24.00	24.00	24.00	24.84	24.84	27.59	23.24
	Median	47.17	46.38	46.38	46.38	46.38	46.38	43.79	45.42	45.85	47.98
	Mean	47.00	45.56	45.69	45.62	45.69	45.69	44.30	46.92	46.85	46.80
	Maximum	70.99	68.36	68.36	68.36	68.36	68.36	61.95	70.99	70.48	66.35
DS4	Minimum	28.13	26.68	23.39	26.68	23.39	23.39	27.68	17.41	**31.42**	21.09
	Median	47.78	46.86	47.38	46.86	47.38	47.38	46.57	46.88	47.12	48.28
	Mean	47.58	47.17	47.31	47.48	47.31	47.31	45.79	46.95	48.14	47.03
	Maximum	74.11	68.36	68.36	68.36	68.36	68.36	64.57	**78.77**	74.55	69.65
DS5	Minimum	28.13	24.84	24.84	24.84	24.84	24.84	27.68	26.24	28.13	28.13
	Median	**49.87**	48.48	48.81	49.17	48.81	48.81	46.10	45.41	46.86	47.12
	Mean	48.61	47.32	47.30	47.36	47.30	47.30	45.64	45.80	48.53	48.12
	Maximum	77.19	74.11	74.11	74.11	74.11	74.11	64.57	74.11	68.36	67.81
DS6	Minimum	26.68	22.57	22.57	22.57	22.57	22.57	24.84	24.84	28.96	28.53
	Median	49.36	44.87	44.87	45.35	44.14	44.87	43.89	47.49	49.43	46.38
	Mean	48.28	45.73	45.81	45.94	45.78	45.85	45.22	47.65	48.80	47.90
	Maximum	71.48	67.81	67.81	68.36	68.36	68.36	67.23	74.11	70.99	70.48

Table 3: AUROC of Different Classifiers

		GAMBoost	RLR	PMR	BGLM	PLR	GLM	SDWD	GBM	GAMLOESS	NB
PIDD	Minimum	75.46	70.31	70.15	70.08	70.15	70.15	69.85	74.00	75.93	70.69
	Median	84.81	83.28	83.15	83.22	83.15	83.15	83.32	84.00	84.38	82.37
	Mean	84.39	83.04	83.04	83.05	83.03	83.04	83.17	83.68	84.08	82.39
	Maximum	92.15	91.41	91.78	91.78	91.70	91.78	92.00	91.11	91.19	91.11
DS1	Minimum	72.77	69.46	69.31	69.38	69.38	69.38	68.77	73.77	75.38	70.23
	Median	84.04	82.74	82.57	82.56	82.56	82.56	82.78	83.61	84.57	83.43
	Mean	83.89	82.52	82.48	82.46	82.48	82.47	82.48	83.75	84.49	82.98
	Maximum	92.52	90.59	90.89	91.19	91.19	91.19	90.96	95.11	95.19	92.44
DS2	Minimum	74.77	68.23	68.31	68.31	68.31	68.31	68.23	74.00	75.00	74.54
	Median	84.98	83.26	83.32	83.32	83.32	83.32	83.25	84.09	84.62	83.89
	Mean	84.53	82.93	82.99	82.99	82.99	82.99	83.09	83.94	84.54	84.20
	Maximum	92.00	91.85	92.15	92.22	92.15	92.15	92.37	91.85	91.70	91.46
DS3	Minimum	73.46	68.92	69.15	69.15	69.15	69.15	69.00	73.38	74.31	73.69
	Median	84.59	82.74	82.63	82.63	82.63	82.63	82.70	83.59	84.70	83.24
	Mean	84.09	82.42	82.44	82.45	82.44	82.44	82.49	83.63	84.28	83.31
	Maximum	93.48	91.48	91.56	91.56	91.56	91.56	91.04	94.52	**95.26**	92.52
DS4	Minimum	74.69	72.00	72.00	72.00	72.08	72.00	71.08	74.92	76.89	71.38
	Median	85.22	82.26	82.37	82.38	82.33	82.37	82.64	84.00	85.06	83.62
	Mean	84.37	82.38	82.39	82.37	82.39	82.39	82.44	84.01	84.82	83.68
	Maximum	92.44	92.59	92.67	92.59	92.67	92.67	93.11	93.19	93.85	94.00
DS5	Minimum	75.00	70.92	71.31	71.31	71.23	71.31	71.46	74.15	**76.92**	76.62
	Median	84.85	82.15	82.27	82.27	82.34	82.27	82.81	84.49	85.08	84.62
	Mean	84.55	82.42	82.45	82.45	82.45	82.45	82.65	84.00	84.75	**84.84**
	Maximum	92.74	93.11	93.11	93.11	93.11	93.11	93.63	92.81	93.41	93.70
DS6	Minimum	73.69	71.54	71.23	71.23	71.23	71.23	71.92	73.85	74.62	75.00
	Median	84.78	81.21	81.17	81.17	81.17	81.17	81.55	84.30	**85.36**	84.96
	Mean	84.27	81.89	81.87	81.87	81.88	81.87	81.93	83.89	84.64	84.30
	Maximum	93.56	92.59	92.89	92.89	92.89	92.89	92.59	93.70	94.30	94.00

Table 4: Sensitivity of Different Classifiers

		GAMBoost	RLR	PMR	BGLM	PLR	GLM	SDWD	GBM	GAMLOESS	NB
PIDD	Minimum	82.00	78.00	78.00	78.00	78.00	78.00	78.00	78.00	74.00	72.00
	Median	88.00	88.00	88.00	88.00	88.00	88.00	90.00	88.00	86.00	84.00
	Mean	87.84	88.68	88.48	88.60	88.52	88.48	89.64	87.36	86.00	84.88
	Maximum	94.00	96.00	96.00	96.00	96.00	96.00	96.00	96.00	96.00	96.00
DS1	Minimum	80.00	76.00	76.00	76.00	76.00	76.00	78.00	76.00	76.00	74.00
	Median	88.00	88.00	88.00	88.00	88.00	88.00	90.00	88.00	88.00	84.00
	Mean	88.84	87.68	87.56	87.44	87.36	87.36	89.40	87.80	86.68	85.40
	Maximum	96.00	96.00	96.00	96.00	96.00	96.00	96.00	96.00	96.00	96.00
DS2	Minimum	80.00	76.00	76.00	76.00	76.00	76.00	82.00	78.00	74.00	76.00
	Median	88.00	88.00	88.00	88.00	88.00	88.00	90.00	86.00	86.00	86.00
	Mean	87.92	88.56	88.36	88.36	88.36	88.36	90.24	86.76	86.2	85.64
	Maximum	94.00	96.00	96.00	96.00	96.00	96.00	96.00	**100**	96.00	96.00
DS3	Minimum	78.00	74.00	74.00	74.00	74.00	74.00	78.00	76.00	74.00	72.00
	Median	88.00	88.00	88.00	88.00	88.00	88.00	90.00	88.00	86.00	84.00
	Mean	87.76	87.48	87.36	87.36	87.36	87.36	89.36	87.44	86.4	83.8
	Maximum	96.00	96.00	96.00	96.00	96.00	96.00	96.00	96.00	96.00	98.00
DS4	Minimum	80.00	78.00	78.00	78.00	78.00	78.00	82.00	76.00	76.00	76.00
	Median	88.00	88.00	88.00	88.00	88.00	88.00	90.00	87.00	86.00	86.00
	Mean	87.92	88.44	88.24	88.32	88.16	88.24	90.16	86.36	85.92	86.80
	Maximum	94.00	98.00	98.00	98.00	98.00	98.00	98.00	96.00	94.00	96.00
DS5	Minimum	80.00	78.00	78.00	78.00	78.00	78.00	88.00	76.00	76.00	76.00
	Median	88.00	88.00	88.00	88.00	88.00	88.00	94.00	86.00	86.00	86.00
	Mean	87.96	88.36	88.24	88.28	88.08	88.24	94.84	86.16	86.4	85.84
	Maximum	94.00	96.00	96.00	96.00	96.00	96.00	**100**	98.00	94.00	92.00
DS6	Minimum	78.00	76.00	76.00	76.00	76.00	76.00	**96.00**	76.00	74.00	74.00
	Median	88.00	88.00	88.00	88.00	88.00	88.00	**100**	87.00	88.00	86.00
	Mean	87.96	87.84	87.52	87.64	87.6	87.52	**99.20**	86.92	87.08	85.32
	Maximum	96.00	98.00	98.00	98.00	98.00	98.00	**100**	96.00	96.00	96.00

Table 5: Specificity of Different Classifiers

		GAMBoost	RLR	PMR	BGLM	PLR	GLM	SDWD	GBM	GAMLOESS	NB
PIDD	Minimum	37.04	37.04	40.74	40.74	40.74	40.74	34.62	33.33	40.74	38.46
	Median	55.56	55.56	55.56	55.56	55.56	55.56	51.85	54.70	59.26	59.26
	Mean	56.26	56.57	56.87	56.79	56.94	56.87	53.28	54.85	59.83	58.33
	Maximum	74.07	76.92	76.92	76.92	76.92	76.92	74.07	80.77	84.62	74.07
DS1	Minimum	34.62	33.33	34.62	34.62	34.62	34.62	33.33	34.62	40.74	33.33
	Median	53.85	55.56	55.56	55.56	55.56	55.56	51.85	55.56	59.26	56.62
	Mean	54.77	54.25	54.84	56.11	56.26	56.26	52.09	56.76	59.45	57.28
	Maximum	77.78	77.78	77.78	81.48	81.48	81.48	70.37	77.78	77.78	76.92
DS2	Minimum	42.31	33.33	38.46	37.04	38.46	38.46	33.33	37.04	42.31	**44.44**
	Median	55.56	55.56	55.56	55.56	55.56	55.56	51.85	57.69	59.26	**62.96**
	Mean	57.38	55.14	55.66	55.59	55.66	55.66	52.31	56.63	59.98	**62.14**
	Maximum	81.48	73.08	74.07	74.07	74.07	74.07	73.08	80.77	80.77	81.48
DS3	Minimum	38.46	34.62	34.62	34.62	34.62	34.62	33.33	34.62	38.46	44.44
	Median	55.56	55.56	55.56	55.56	55.56	55.56	51.85	59.26	59.26	62.96
	Mean	57.15	55.96	56.25	56.18	56.25	56.25	52.02	57.44	58.79	62.23
	Maximum	77.78	77.78	77.78	77.78	77.78	77.78	70.37	80.77	84.62	81.48
DS4	Minimum	42.31	38.46	38.46	38.46	38.46	38.46	34.62	33.33	40.74	38.46
	Median	55.56	55.56	57.69	58.48	58.48	57.69	51.85	59.26	59.26	59.26
	Mean	57.30	55.98	56.86	56.94	56.94	56.86	52.69	58.74	60.90	58.48
	Maximum	85.19	80.77	80.77	80.77	80.77	80.77	76.92	80.77	85.19	81.48
DS5	Minimum	42.31	38.46	38.46	38.46	38.46	38.46	7.41	37.04	42.31	42.31
	Median	56.62	57.69	57.69	57.69	57.69	57.69	33.33	59.26	59.26	59.26
	Mean	58.64	56.71	56.86	56.86	56.86	56.86	33.19	58.49	60.65	61.02
	Maximum	85.19	81.48	81.48	81.48	81.48	81.48	51.85	80.77	81.48	81.48
DS6	Minimum	38.46	34.62	34.62	34.62	34.62	34.62	0.00	40.74	40.74	42.31
	Median	58.48	55.56	55.56	55.56	55.56	55.56	5.63	59.26	59.26	62.96
	Mean	58.28	55.81	56.25	56.18	55.95	56.25	5.97	59.15	59.98	61.40
	Maximum	81.48	74.07	77.78	77.78	77.78	77.78	18.52	**85.19**	77.78	77.78

Table 6: Logloss of Different Classifiers

		GAMBoost	RLR	PMR	BGLM	PLR	GLM	SDWD	GBM	GAMLOESS	NB
PIDD	Minimum	37.18	37.94	38.48	37.71	37.64	37.62	43.58	37.66	36.31	35.24
	Median	46.42	48.17	48.17	48.10	48.11	48.11	50.57	46.89	46.92	56.27
	Mean	46.77	48.50	48.52	48.50	48.51	48.52	50.41	47.50	47.95	55.93
	Maximum	58.26	62.50	61.90	62.78	62.81	62.97	59.13	57.96	69.86	81.20
DS1	Minimum	36.49	38.47	38.06	38.14	38.06	38.06	44.19	35.52	33.41	34.02
	Median	47.05	48.90	48.94	48.93	48.94	48.94	50.96	47.67	46.13	49.88
	Mean	47.09	49.05	49.05	49.04	49.04	49.05	50.98	47.33	47.07	51.56
	Maximum	57.86	64.40	64.77	64.56	64.76	64.77	60.26	57.44	72.65	72.77
DS2	Minimum	36.22	38.49	37.57	37.66	37.61	37.57	43.47	36.39	34.63	35.30
	Median	46.00	48.06	48.04	48.00	48.04	48.04	50.38	47.00	46.43	49.54
	Mean	46.53	48.59	48.59	48.57	48.58	48.59	50.41	47.14	47.03	48.60
	Maximum	58.01	64.69	65.21	64.95	65.20	65.21	59.61	57.48	70.87	66.84
DS3	Minimum	36.36	37.88	37.31	37.42	37.31	37.31	44.03	35.95	34.25	34.72
	Median	46.90	48.94	48.93	48.93	48.93	48.93	50.97	47.54	46.27	49.58
	Mean	46.82	49.07	49.07	49.06	49.07	49.07	50.96	47.47	47.21	49.55
	Maximum	58.17	64.65	65.14	64.91	65.14	65.14	60.13	57.88	73.07	64.11
DS4	Minimum	36.77	37.78	37.28	37.38	37.32	37.28	43.48	35.52	34.97	**30.98**
	Median	45.59	49.11	49.14	49.13	49.13	49.14	51.43	46.62	45.82	51.38
	Mean	**46.68**	49.43	49.44	49.42	49.43	49.44	51.25	47.17	46.82	51.29
	Maximum	**56.83**	60.79	61.26	61.14	61.10	61.26	58.40	57.78	71.40	73.04
DS5	Minimum	36.07	37.37	36.81	36.92	36.84	36.81	43.31	34.93	34.61	32.13
	Median	45.79	49.38	49.40	49.39	49.39	49.40	51.49	46.44	45.71	47.87
	Mean	46.43	49.42	49.43	49.42	49.43	49.43	51.20	47.06	46.76	47.26
	Maximum	57.40	61.55	62.05	61.90	62.04	62.05	58.45	58.52	71.76	63.24
DS6	Minimum	36.15	37.87	37.45	37.56	37.45	37.45	44.24	36.39	34.79	33.94
	Median	46.69	49.95	50.06	50.04	50.06	50.06	51.96	46.49	**45.58**	46.42
	Mean	46.61	49.72	49.72	49.72	49.68	49.72	51.56	47.13	46.66	47.07
	Maximum	57.53	61.76	62.14	62.00	62.14	62.14	58.90	57.58	73.71	61.63

Table 7: Average Best Classification Results for Different Dataset

		Accuracy	Kappa	AUROC	Sensitivity	Specificity	Logloss
PIDD	Minimun						
	Median						
	Mean						
	Maximum						
DS1	Minimun						
	Median						
	Mean						
	Maximum						
DS2	Minimun					38.52	
	Median						
	Mean			83.52			
	Maximum						
DS3	Minimun						
	Median						
	Mean						
	Maximum						
DS4	Minimun						
	Median					57.31	
	Mean					57.17	
	Maximum					81.34	
DS5	Minimun	68.15	26.25	73.02	78.60		36.58
	Median	77.45	47.94	83.74			
	Mean	77.27	47.33				
	Maximum	87.53	72.26				61.90
DS6	Minimun						
	Median				88.90		
	Mean				88.46		
	Maximum			93.23	97.40		
